# Research of text paraphrase generation based on self-contrastive learning

**DOI:** 10.1371/journal.pone.0327613

**Published:** 2025-09-02

**Authors:** Ling Yuan, Hai Ping Yu, Junlin Ren, Ping Sun

**Affiliations:** 1 School of Computing Science and Technology, Huazhong University of Science and Technology, Wuhan, Hubei, People’s Republic of China; 2 Wuhan Vocational College of Software and Engineering (Wuhan Open University), Wuhan, Hubei, People’s Republic of China; Philadelphia University, JORDAN

## Abstract

The goal of this study is to improve the quality and diversity of text paraphrase generation, a critical task in Natural Language Generation (NLG) that requires producing semantically equivalent sentences with varied structures and expressions. Existing approaches often fail to generate paraphrases that are both high-quality and diverse, limiting their applicability in tasks such as machine translation, dialogue systems, and automated content rewriting. To address this gap, we introduce two self-contrastive learning models designed to enhance paraphrase generation: the Contrastive Generative Adversarial Network (ContraGAN) for supervised learning and the Contrastive Model with Metrics (ContraMetrics) for unsupervised learning. ContraGAN leverages a learnable discriminator within an adversarial framework to refine the quality of generated paraphrases, while ContraMetrics incorporates multi-metric filtering and keyword-guided prompts to improve unsupervised generation diversity. Experiments on benchmark datasets demonstrate that both models achieve significant improvements over state-of-the-art methods. ContraGAN enhances semantic fidelity with a 0.46 gain in BERTScore and improves fluency with a 1.57 reduction in perplexity. In addition, ContraMetrics achieves gains of 0.37 and 3.34 in iBLEU and P-BLEU, respectively, reflecting greater diversity and lexical richness. These results validate the effectiveness of our models in addressing key challenges in paraphrase generation, offering practical solutions for diverse NLG applications.

## 1 Introduction

Natural Language Generation (NLG) [1,2] is a pivotal component of Natural Language Processing (NLP), with broad applications in automated news writing [3,4], subtitle generation, and intelligent customer service. NLG transforms abstract conceptual representations into coherent text [5,6], and neural network modeling has emerged as its primary technical approach.

Among NLG tasks, text paraphrase generation stands out as a foundational challenge, aiming to rephrase input sentences into alternative forms that preserve semantic equivalence. This task plays a critical role in applications such as question answering [7,8], machine translation [9,10], and semantic analysis [11,12]. Additionally, it serves as a key method for textual data augmentation [13,14], enhancing dataset diversity and improving the robustness of NLP systems.

Despite its importance, the task of paraphrase generation remains inherently complex, as it requires achieving a delicate balance between maintaining semantic fidelity and generating outputs with sufficient lexical and syntactic variation. While numerous methods have been proposed, they are often limited in scope and effectiveness when faced with the diverse and dynamic requirements of real-world applications. Existing models tend to focus narrowly on specific aspects of the task, resulting in suboptimal performance and a lack of generalizability.

These limitations are particularly evident in three key areas:

1. **Trade-off between Discrepancy and Semantic Fidelity**: Current models struggle to balance producing outputs that differ sufficiently from the input while maintaining semantic integrity. Methods prioritizing semantic fidelity often generate outputs overly similar to the original sentence [15], while those focusing on lexical or syntactic variation risk introducing semantic errors [16].
2. **Insufficient Generative Diversity**: Many models generate a single paraphrase per input or rely on template-based transformations, which limit output variability and fail to meet the demands of large-scale applications. Ensuring diverse, meaningful paraphrases remains an open challenge.
3. **Model Degradation and Bias**: Generative models frequently suffer from lexical frequency imbalances and anisotropic embedding spaces, resulting in repetitive or incoherent outputs. This phenomenon, known as representation degradation, undermines the quality and reliability of generated texts [17].

Existing solutions fail to adequately address these challenges due to reliance on heuristic approaches or limited modeling techniques. Moreover, the lack of robust methods for unsupervised paraphrase generation further restricts their applicability in resource-constrained scenarios.

To bridge these gaps, this paper introduces a novel approach based on self-contrastive learning, addressing both supervised and unsupervised paraphrase generation tasks. Specifically, we propose two algorithmic models:

**Contrastive Generative Adversarial Network (ContraGAN)**: Designed for supervised scenarios, ContraGAN employs a learnable discriminator within a GAN framework, utilizing pseudo-labeling for self-contrastive learning to enhance semantic fidelity and diversity.

**Contrastive Model with Metrics (ContraMetrics)**: Tailored for unsupervised scenarios, ContraMetrics combines multi-metric filtering and keyword-guided prompts to identify high-quality samples, enabling contrastive learning without requiring extensive parallel datasets.

Extensive experiments demonstrate that ContraGAN and ContraMetrics address key limitations of prior works, achieving superior performance in fluency, diversity, and semantic fidelity. By bridging the gap between supervised and unsupervised paraphrase generation, our methods offer practical and scalable solutions for a wide range of NLP applications.

This paper makes several contributions to the field of text paraphrase generation:

1. By integrating techniques such as generative adversarial networks [18,19] and contrastive learning [20], a novel self-contrastive learning-based method is proposed.
2. The self-contrastive learning method is applied to both supervised and unsupervised scenarios through the development of the ContraGAN and ContraMetrics models, respectively.
3. Comparative experiments were conducted to demonstrate the superiority of the proposed models over existing benchmark models. In addition, ablation experiments were performed to evaluate the impact of the self-contrastive learning method on model performance.

The structure of this paper is as follows: the introduction outlines the study’s background, motivation, and contributions. Sect 2 presents the self-contrastive learning approach addressing current text generation challenges. Sect 3 details the ContraGAN model for supervised learning, while Sect 4 covers the ContraMetrics model for unsupervised learning. Sect 5 reports the experimental results and analysis. Lastly, Sect 6 summarizes the findings and contributions.

## 2 Analysis of paraphrase generation based on self-contrastive learning

### 2.1 Issues of text generation paradigms

The text generation paradigm in NLP is based on autoregressive prediction for decoding generation. However, according to the paradigm, several key issues can be identified in the generation task:

1. **Exposure Bias:** Seq2Seq models are trained on labeled sequences $\langle y_{1},\ldots ,y_{t-1}\rangle$, but generate sequences $\langle {\hat{y}}_{1},\ldots ,{\hat{y}}_{t-1}\rangle$ during inference, leading to a distribution mismatch known as exposure bias [21].
2. **Lack of Diversity:** Maximum likelihood estimation (MLE) training aligns model outputs with specific labels, enhancing accuracy but limiting generative diversity due to local optimization.
3. **Model Degradation:** In text paraphrase tasks, the non-uniform distribution of language units causes anisotropy in the embedding space, frequently sampling high-occurrence units, even when contextually incoherent.

To tackle the limitations in existing text generation paradigms, we introduce self-contrastive learning, which extends traditional contrastive learning by leveraging self-generated samples as both positive and negative pairs. This approach optimizes the model’s vector space, enabling it to capture subtle semantic differences even in minor input variations. The proposed self-contrastive learning framework addresses key challenges as follows:

1. Mitigating Exposure Bias: By generating positive and negative pairs directly from model outputs, the training distribution aligns more closely with the inference phase, reducing discrepancies caused by exposure bias.
2. Enhancing Generative Diversity: The use of multiple positive and negative pairs transforms the learning objective into a multi-target optimization problem, encouraging the model to produce diverse outputs through a customized loss function.
3. Combating Model Degradation: Sampling from densely populated output distributions identifies more challenging pairs, refining vector space representation and improving the model’s consistency and robustness.

### 2.2 Self-contrastive learning

To tackle the limitations in existing text generation paradigms, we introduce self-contrastive learning, which extends traditional contrastive learning by leveraging self-generated samples as both positive and negative pairs. This approach optimizes the model’s vector space, enabling it to capture subtle semantic differences even in minor input variations. The proposed self-contrastive learning framework addresses key challenges as follows:

1. Mitigating Exposure Bias: By generating positive and negative pairs directly from model outputs, the training distribution aligns more closely with the inference phase, reducing discrepancies caused by exposure bias.
2. Enhancing Generative Diversity: The use of multiple positive and negative pairs transforms the learning objective into a multi-target optimization problem, encouraging the model to produce diverse outputs through a customized loss function.
3. Combating Model Degradation: Sampling from densely populated output distributions identifies more challenging pairs, refining vector space representation and improving the model’s consistency and robustness.

### 2.3 Text paraphrase generation based on self-contrastive learning methods

**Fig 1:**
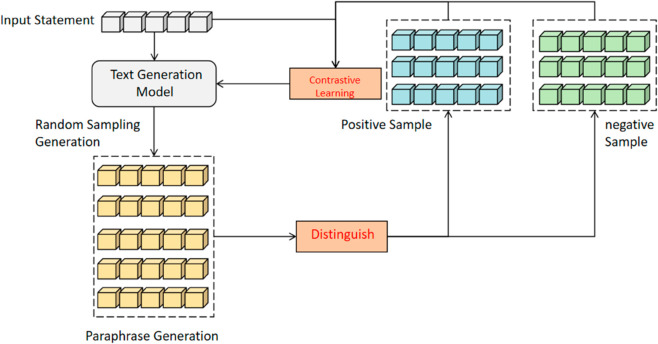
Illustration of paraphrase generation method based on self-contrastive learning.

Self-contrastive learning methods offer a theoretical solution to the challenges in text paraphrase generation. The key to self-contrastive learning lies in differentiating self-generated samples to identify corresponding positive and negative examples. As shown in Fig 1, for a given input, the text generation model randomly samples multiple paraphrases, which are then categorized as positive or negative based on specific differentiation criteria. The model is subsequently refined using contrastive learning techniques.

Models are developed to address the task of text paraphrase generation in both supervised and unsupervised learning contexts. Both models are grounded in a unified self-contrastive learning approach but are implemented with distinct methods, detailed in Sects 3 and 4, respectively.

## 3 ContraGAN for supervised text paraphrase generation

In this paper, we introduce ContraGAN, a generative adversarial network that leverages data as a conduit and contrastive learning as its training objective. By removing the gradient flow dependency between discriminator and generator, ContraGAN circumvents traditional GAN limitations in natural language processing. This GAN framework enriches paraphrase generation by enabling the discriminator to iteratively identify challenging positive and negative samples, thereby amplifying the benefits of contrastive learning for improved paraphrase generation performance.

### 3.1 ContraGAN model structure

**Fig 2:**
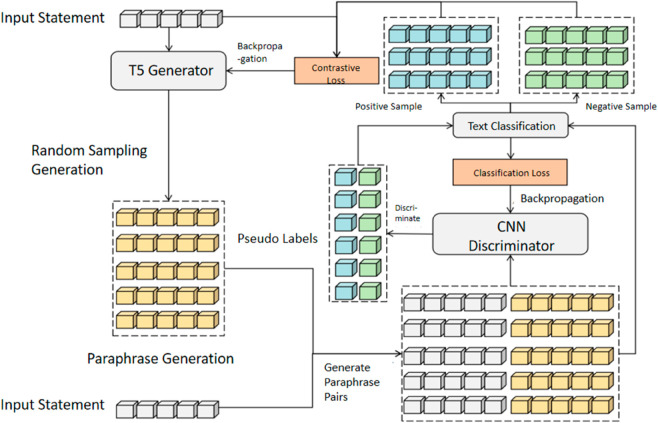
Illustration of ContraGAN model structure.

ContraGAN introduces a novel architecture combining a T5-based generator and a CNN-based discriminator [22]. The generator leverages a pre-trained T5 model in an encoder-decoder framework to produce multiple paraphrase samples, which are concatenated with the input to form paraphrase pairs. These pairs are evaluated by the CNN discriminator, which assigns pseudo-labels indicating their likelihood as true paraphrases. The generator employs contrastive learning to refine semantic representation, while the discriminator enhances robustness through binary cross-entropy loss. This collaborative mechanism, illustrated in Fig 2, enables ContraGAN to align generation and evaluation seamlessly, addressing challenges in generative diversity and semantic fidelity.

### 3.2 Contrastive learning training process

**Fig 3:**
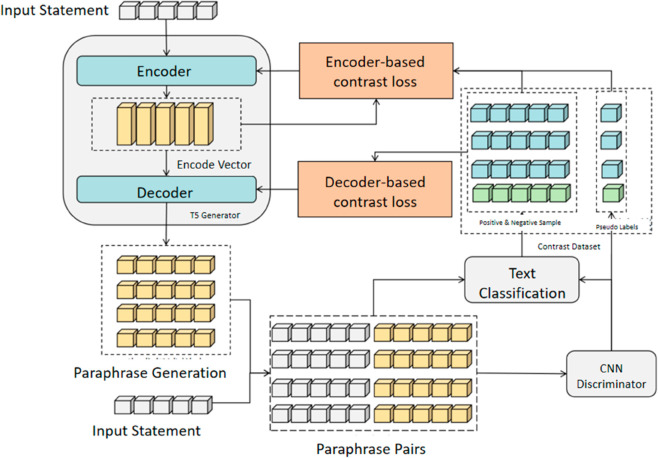
Illustration of ContraGAN generator training. The discriminator outputs a pseudo-label.

The contrastive learning training process of ContraGAN involves two distinct phases: generator training and discriminator training. This approach employs contrastive learning to leverage the relationships between positive and negative samples. The generator and discriminator are trained independently,the process for generator training begins with the T5 generator producing a set of paraphrased samples based on the input statements. These generated samples are then paired with the original input to create paraphrase pairs, which are evaluated by the CNN discriminator. These classifications, combined with the pseudo-labels, form a contrastive dataset. The T5 generator then applies contrastive learning, using both encoder and decoder-based contrastive loss calculations to update its parameters. The process is shown in Fig 3.

The encoder’s parameters are optimized using contrastive loss from positive and negative samples, while the decoder’s parameters are updated based on the loss from positive samples and encoded vectors. Paraphrase pairs are generated in batches, then utilized by the CNN discriminator to construct the contrastive dataset, enabling effective contrastive learning.

The generator initially produces a substantial number of samples during the training phase. These samples are then evaluated by the discriminator to construct a contrastive dataset. In the inference phase, the generator produces multiple output sequences ${\hat{Y}}_{ik}$ based on the input *X*_*i*_. The discriminator *D* evaluates the pairs $\left(X_{i},{\hat{Y}}_{i1}),(X_{i},{\hat{Y}}_{i2}),\ldots ,(X_{i},{\hat{Y}}_{iK}\right)$ against a classification threshold *T*, thereby categorizing them into positive and negative sample sets, *P* and *N*, respectively. This process ultimately constructs the contrastive dataset $\hat{S}$.

$$P=<X_{i},{\left(Y\right)}_{i}>,D\left(X_{i},{\left(Y\right)}_{i}\right)>T$$ (1)

$$Q=<X_{j},{\left(Y\right)}_{j}>,D\left(X_{j},{\left(Y\right)}_{j}\right)\leq T$$ (2)

$$\overset{ˇ}{S}=\{X_{i},Y_{i},Y_{i}^{+},Y_{i}^{-},DIS(X_{i},{\overset{ˇ}{Y}}_{i}),DIS(X_{j},{\overset{ˇ}{Y}}_{j})\}$$ (3)

**Fig 4:**
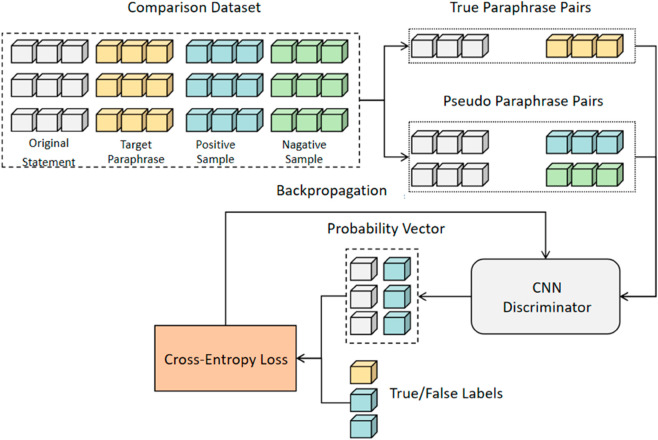
Illustration of ContraGAN discriminator training.

Based on dataset $\overset{ˇ}{S}$, the discriminator outputs a two-dimensional tensor representing the probability that an input sample pair is either a generated or actual paraphrase. The cross-entropy loss is calculated using this probability vector and the true/false labels. $\overset{ˇ}{S}$ consists of each sample *X*_*i*_, its target paraphrase *Y*_*i*_, and multiple positive ($Y_{i}^{+}$) and negative ($Y_{i}^{-}$) samples. The discriminator is trained to classify $\left(X_{i},Y_{i}\right)$ as positive and $\left(X_{i},Y_{i}^{+}\right)$, $\left(X_{i},Y_{i}^{-}\right)$ as negative, as shown in Fig 4.

### 3.3 ContraGAN-based text paraphrase generation

**Fig 5:**
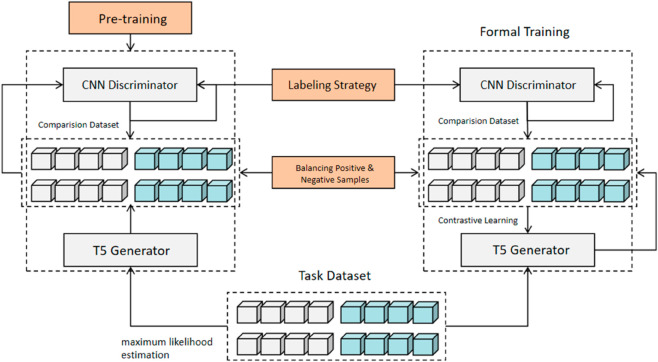
Illustration of complete training process that incorporates strategies.

Key strategies for stable training and improving paraphrase quality include pre-training, balancing positive and negative samples, and label smoothing. As shown in Fig 5, the T5 generator is first pre-trained with maximum likelihood estimation, followed by training the CNN discriminator with label smoothing. These pre-trained components are then used in the formal training phase to generate high-quality paraphrases.

The training process is outlined in Algorithm 1. ContraGAN uses self-contrastive learning for paraphrase generation with a pre-trained T5 generator and CNN-based discriminator. The model alternates between generator and discriminator training, with positive and negative samples created based on a threshold. The generator’s parameters are updated using contrastive losses to balance semantic fidelity and generative diversity. Label smoothing and pseudo-labeling stabilize training and enhance paraphrase quality, addressing exposure bias.

**Algorithm 1 ContraGAN training process algorithm.**

**Input:** Dataset S={X,Y}, number of samples *n*, generator learning rate *lr*_*G*_, generator training steps *g*, discriminator learning rate *lr*_*D*_, discriminator training steps *d*, number of training epochs *e*, threshold *T*, loss weighting factor *α*, label smoothing parameter *ε*

**Output:** Text paraphrase generation model ContraGAN

1: Use the pre-trained T5 model as the generator *G*, initialize the CNN discriminator *D*;

2: Pre-train the generator *G* on *S* based on maximum likelihood estimation;

3: Use *G* to sample generated samples, sample real samples on *S*, and pre-train the discriminator *D*;

4: **for** *e* steps **do**

5:   Freeze the generator;

6:   Sample output $\leftarrow \{{\overset{ˇ}{Y}}_{1},{\overset{ˇ}{Y}}_{2},\ldots ,{\overset{ˇ}{Y}}_{n}\}=G(X)$;

7:   Positive and negative sample discrimination $\leftarrow p_{i}=D(X,{\overset{ˇ}{Y}}_{i}),P_{j}=\{Y_{j},p_{j}>T\},N_{k}=\{Y_{k},p_{k}<T\}$;

8:   Construct comparison dataset using discriminator $\leftarrow \overset{ˇ}{S}=\{X,Y,Y^{+},Y^{-},p\}$;

9:   Unfreeze the generator;

10:   **for** *g* steps **do**

11:    Encoding vectors *H*, *H*_*P*_, and *H*_*N*_ $\leftarrow G_{E}(X),G_{E}(P),G_{E}(N)$;

12:    Encoder contrastive loss $\leftarrow L_{Encoder}(H,H_{P},H_{N},p)$;

13:    Decoder contrastive loss $\leftarrow L_{Decoder}(H,P,p)$;

14:    Overall loss $\leftarrow L=\alpha \cdot L_{Encoder}+(1-\alpha )\cdot L_{Decoder}$;

15:    Update model parameters $\leftarrow lr_{G}\cdot \frac{\partial L}{\partial \theta }$;

16:   **end for**

17:   Freeze the generator, unfreeze the discriminator;

18:   **for** *d* steps **do**

19:    Perform paraphrase classification on $\overset{ˇ}{S}$ $\leftarrow \overset{ˇ}{C}=D(X,Y)$;

20:    Update the discriminator $\leftarrow lr_{D}$ ⋅ $\frac{\partial }{\partial \theta }Loss(C,\overset{ˇ}{C})$, $\text{where }C=\left\{ \begin{array}{cc} \varepsilon & \text{if }Y\in \overset{ˇ}{Y}\\ 1-\varepsilon & \text{otherwise} \end{array}\right.$;

21:   **end for**

22:   Freeze the discriminator;

23: **end for**

### 3.4 ContraGAN theoretical analysis

Encoder-based and Decoder-based Contrastive Loss: The encoder-based contrastive loss computes the similarity between the original utterance and the encoded vectors of positive and negative samples, with time complexity *O*(*n*^2^*d*  +  *nd*^2^) where *n* is the number of units in the input utterance, and *d* is the feature vector dimension. Similarly, the decoder-based contrastive loss, based on maximum likelihood estimation across multiple positive samples, exhibits the same complexity. However, due to the inability to perform batch-based gradient updates, gradient computation must be performed individually for each input sample, making training nearly *b* times slower compared to conventional batch size *b* methods.

Key Innovations in ContraGAN: Despite these challenges, ContraGAN introduces several innovations:

1. **Alignment of Training and Inference Distributions**: The self-contrastive framework eliminates exposure bias by aligning input distributions during training and inference.
2. **Optimized Vector Space Representation**: By utilizing multiple self-sampled positive and negative examples, ContraGAN refines vector positioning based on semantic distances, creating an oriented and uniform vector space. This mitigates degradation typically observed in maximum likelihood estimation.
3. **Enhanced Generative Diversity**: Decoder-based contrastive learning, leveraging multiple positive samples, reduces repetitive outputs and promotes diverse generation.

ContraGAN surpasses prior methods by addressing multi-objective optimization, enabling greater diversity while simplifying GAN training. Strategies such as balanced sample selection, pre-training, and robust labeling further ensure stable training and minimize noise. Deep neural networks and attention mechanism are utilized for document feature extraction. The label distribution-based correlation residual network is introduced to mitigate training costs and network degradation. It annotates text sequences with a relevant label subset and uses related knowledge to discern label correlations, enhancing the classification probability of relevant labels and reducing the irrelevant ones.

## 4 ContraMetrics for unsupervised text paraphrase generation

In response to the limited availability of text paraphrase datasets—particularly in Chinese and specialized fields—this study proposes an unsupervised learning approach for text paraphrase generation using a metric-based comparison learning model, called ContraMetrics. Unlike ContraGAN, which relies on conventional adversarial methods, ContraMetrics generates positive and negative samples through a metric-driven mining strategy. To enhance performance in unsupervised settings, a keyword prompt-based transfer learning technique is employed, transferring paraphrase capabilities from general to task-specific domains during pre-training. The combination of metric-driven sampling and keyword transfer constitutes ContraMetrics’ approach to paraphrase generation.

### 4.1 ContraMetrics model structure

**Fig 6:**
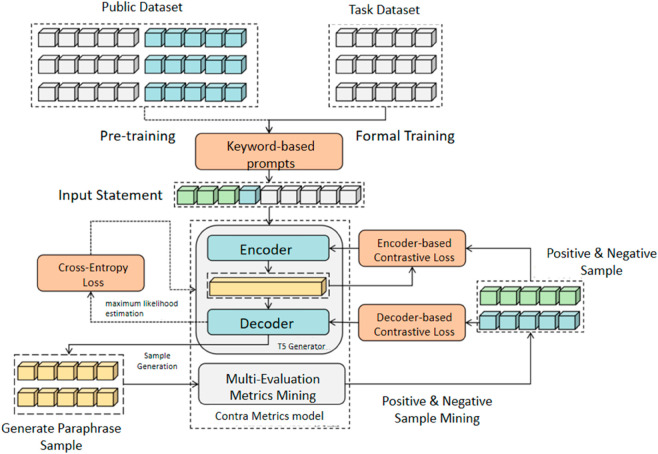
Illustration of ContraMetrics model structure.

**Fig 7:**
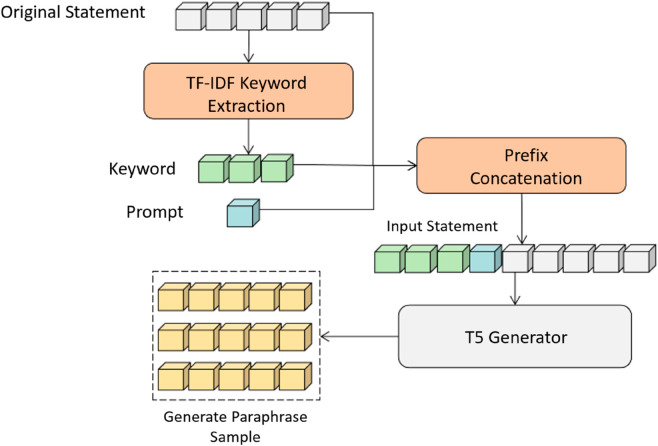
Illustration of Keyword-based prompt learning.

The ContraMetrics architecture is shown in Fig 6. The dataset consists of raw text *X*, without target paraphrases, learned from bootstrapped metric-based samples. Using the same pre-trained T5 generator as ContraGAN, ContraMetrics pre-trains on public parallel datasets (dotted line, Fig 7) via maximum likelihood estimation with cross-entropy loss. Formal training on task-specific datasets (solid line Fig 7) follows, leveraging mined positive and negative samples through multi-metric evaluation. Keyword-based prompts are used in both phases to refine inputs and transfer model capabilities to task-specific datasets.

### 4.2 Transfer learning based on keyword prompts

To enhance performance in unsupervised learning, pre-training on a public paraphrasing dataset *S*_1_ with keyword-based prompts is employed. Prompts modify the input *X* to align with the task, leveraging large language models’ semantic understanding for adaptive output.

In the first stage, the T5 model is trained on paraphrase pairs (*X*,*Y*) from *S*_1_ using a *Prompt* as a prefix. This trains the model to generate paraphrases aligned with the task. In the second stage, the model is fine-tuned on the task-specific dataset *S*_2_, which contains only text *X*. Using the same *Prompt* ensures transfer learning consistency. Using the same *Prompt* as a prefix, the model will output in the same way even if the datasets are different.

To preserve semantic content, contextual information is added to the prompt. Keywords extracted via TF-IDF from *X* are appended to the *Prompt*, combining task instructions with critical semantics. For instance, the input “The weather in Beijing is nice today” with the prompt “Beijing” becomes “Beijing, Text Repetition: The weather is nice today!” This approach ensures effective transfer learning and contextually appropriate paraphrase generation.

### 4.3 ContraMetrics-based text paraphrase generation

The ContraMetrics training process, shown in Algorithm 2, consists of two stages. First, supervised learning with TF-IDF-based keyword prompts fine-tunes a pre-trained T5 model. In the unsupervised phase, paraphrases generated from a task-specific dataset are evaluated using performance metrics to mine positive and negative samples. Contrastive learning with a weighted loss function refines the model, enhancing paraphrase quality and diversity.

**Algorithm 2 ContraMetrics training process algorithm.**

**Input:** Public dataset *S*_1_ = {*X*,*Y*}, task dataset *S*_2_ = {*X*}, prompt *Prompt*, number of samples *n*, learning rate *lr*, contrastive steps *t*, training epochs *e*, positive sample threshold *T*_*P*_, negative sample threshold *T*_*N*_, loss weighting factor *α*

**Output:** Text paraphrase generation model ContraMetrics

1: Initialize the pre-trained T5 model weights *G*;

2: Supervised pre-training on public dataset *S*_1_:   Extract keywords based on TF-IDF K=TF-IDF(X);   Perform maximum likelihood estimation training with keyword prompts $\leftarrow \overset{ˇ}{Y}=G(K+Prompt+X)$;

3: Unsupervised training on task dataset *S*_2_:

4: **for** *e* steps **do**

5:   Extract keywords based on TF-IDF K=TF-IDF(X);

6:   Generate paraphrases based on keyword prompts $\leftarrow \overset{ˇ}{X}=G(K+Prompt+X)$;

7:   Generate sampled paraphrases $\left\{Y_{1},Y_{2},\ldots ,Y_{n}\}\leftarrow G(\overset{ˇ}{X};n\right)$;

8:   Calculate evaluation metric scores $S_{i1},S_{i2},\ldots ,S_{in}$ $\leftarrow S_{i}(X,Y,Y_{1},Y_{2},\ldots ,Y_{n};n)$;

9:   Mine positive and negative samples for each metric $\leftarrow P_{j}=\{Y_{j},S_{j}>T_{P}\},N_{k}=\{Y_{k},S_{k}<T_{N}\}$;

10:   Perform voting to select positive samples $P:\{P_{1},P_{2},\ldots ,P_{J}\}$ and negative samples $N:\{N_{1},N_{2},\ldots ,N_{K}\}$;

11:   **for** *t* steps **do**

12:    Encoding vectors *H*, $H_{P_{j}}$, and $H_{N_{k}}$ $\leftarrow G_{E}(X),G_{E}(P_{j}),G_{E}(N_{k})$;

13:    Encoder loss $\leftarrow L_{Encoder}(H,H_{P_{j}},H_{N_{k}})$;

14:    Decoder loss $\leftarrow L_{Decoder}(H,P)$;

15:    Overall loss $\leftarrow L=\alpha \cdot L_{Encoder}+(1-\alpha )\cdot L_{Decoder}$;

16:    Update model parameters $\leftarrow lr\cdot \frac{\partial L}{\partial \theta }$;

17:   **end for**

18: **end for**

The ContraMetrics model employs a T5 generative model based on an encoder-decoder framework, similar to ContraGAN but without deterministic target paraphrasing or discriminator-based pseudo-labeling. This simplifies the loss function for contrastive learning.

For an input statement *X*_*i*_, the encoding vector is *H*_*i*_, with $H_{P_{i}}$ and $H_{N_{i}}$ representing positive and negative sample encodings. Encoder-based contrastive learning uses a multi-objective cross-entropy loss to measure the similarity between *H*_*i*_, $H_{P_{i}}$, and $H_{N_{i}}$, as shown in Eq (4). Here, *S* is the cosine similarity, and $H_{P_{i}}^{j}$ and $H_{N_{i}}^{k}$ are the *j*-th positive and *k*-th negative sample encodings.

Decoder-based contrastive learning calculates the loss *L* directly from the positive sample set $P=\{P_{1},P_{2},\ldots ,P_{j}\}$, as in Eq (5). The encoder loss is averaged across positive samples, and the overall loss function follows the format in Eq (6).

$$\;L_{\text{Encoder}}=-\frac{1}{n}\sum_{i=1}^{n}\log\frac{\sum_{j=1}^{J}e^{S(H_{i},H_{P_{i}}^{j})}}{\sum_{j=1}^{J}e^{S(H_{i},H_{P_{i}}^{j})}+\sum_{k=1}^{K}e^{S(H_{i},H_{N_{i}}^{k})}}$$ (4)

$$\;L_{\text{Decoder}}=-\frac{1}{n}\sum_{i=1}^{n}\frac{1}{J}\sum_{j=1}^{J}L(X_{i},P_{ij})$$ (5)

$$\;L=\alpha \cdot L_{\text{Encoder}}+(1-\alpha )\cdot L_{\text{Decoder}}$$ (6)

### 4.4 ContraMetrics theoretical analysis

The ContraMetrics model introduces several novel strategies: self-comparison learning, keyword-based prompting, pre-training transfer, and multi-metric mining. By leveraging a pre-trained T5 model, initially trained on public datasets, ContraMetrics seamlessly transitions to unsupervised task-specific training. This pre-training transfer allows the model to apply its supervised rephrasing capabilities to unsupervised tasks, enhancing the semantic accuracy of generated paraphrases.

A key innovation is the use of TF-IDF-based keyword inputs, which enrich the model with task-relevant semantic features, boosting both fluency and semantic fidelity in paraphrase generation. ContraMetrics also integrates multi-metric evaluation for sample selection, allowing it to mine positive and negative samples based on semantic fidelity, fluency, and discrepancy, without requiring labeled data. This voting-based mechanism further ensures high-quality paraphrases by filtering extreme cases, dramatically improving model robustness and quality.

## 5 Experimental results

### 5.1 Experiment settings

#### 5.1.1 Datasets.

To address the limited research on text paraphrase generation in the Chinese language domain, the model’s performance is validated using four major Chinese-language datasets: LCQMC [23], Phoenix, BQ-Corpus [24] and PAWS-X [25], thereby contributing to the advancement of research in this area.

The LCQMC dataset, developed by the Harbin Institute of Technology (HIT), is a Chinese question-matching corpus from the Baidu Knowledge domain, used for binary classification. A label of 1 indicates semantic similarity, while 0 denotes dissimilarity. For this work, only samples labeled as 1 were retained. The processed LCQMC dataset statistics are shown in Table 1.

**Table 1:** LCQMC dataset statistics.

LCQMC	Training Set	Validation Set	Test Set
Sample size	138563	3249	4401
Input average length	10.04	9.73	13.33
Output average length	11.23	10.88	14.44
Maximum length	40	28	37
Minimum length	2	4	10

The Phoenix dataset, developed by Baidu, comprises short text pairs from the business domain, extracted from search logs and paraphrased with 95% accuracy. It primarily features questions and offers a large volume of data, used in supervised experiments. Dataset statistics are shown in Table 2.

**Table 2:** Phoenix dataset statistics.

Phoenix	Training Set	Validation Set	Test Set
Sample size	449999	9999	42607
Input average length	8.58	8.58	8.57
Output average length	9.50	9.51	9.50
Maximum length	33	29	29
Minimum length	3	3	3

The BQ-Corpus, a large-scale dataset in the banking domain, is designed for question matching tasks. Derived from online banking logs, it is used in unsupervised experiments, retaining only samples labeled 1. Dataset statistics are shown in Table 3.

**Table 3:** BQ-Corpus dataset statistics.

BQ-Corpus	Training Set	Validation Set	Test Set
Sample size	49999	4999	9998
Input average length	11.55	11.61	11.65
Maximum length	154	130	101
Minimum length	1	2	2

PAWS-X, developed by Google, is a paraphrase dataset featuring longer texts with specialized knowledge and technical terms. Used in unsupervised experiments, only samples labeled 1 are retained. Dataset statistics are shown in Table 4.

**Table 4:** PAWS-X dataset statistics.

PAWS-X	Training Set	Validation Set	Test Set
Sample size	21828	851	1998
Input average length	44.34	42.09	43.24
Maximum length	150	102	120
Minimum length	7	10	2

#### 5.1.2 Benchmark models.

This study develops ContraGAN and ContraMetrics using the basic T5 model [26] trained with the Mengzi framework [27]. Pre-training incorporates adversarial learning and dynamic fine-tuning for robustness. Formal training excludes additional strategies to ensure a clear evaluation of the proposed self-comparative learning approach.

For supervised scenarios, benchmark models include T5, LaserTagger [15], FELIX [28], BART [29]and HRQ-VAE [30] , with experiments conducted on the LCQMC and Phoenix datasets.

In unsupervised scenarios, the SeqGAN [31], DPGAN [32], DSS-VAE [33], PEGASUS [34] and STVAE [35] models serve as benchmarks, with experiments carried out on the BQ-Corpus and PAWS-X datasets.

#### 5.1.3 Evaluation indicators.

Model-generated paraphrases are evaluated on semantic fidelity, fluency, and generative diversity, which includes variation and richness. Semantic fidelity, crucial for maintaining deep semantic alignment, is assessed using BERTScore [36]. This method encodes paraphrases and inputs with BERT, computing similarity scores based on vector representations to reflect semantic congruence.

$$H_{X}=BERT\left(X\right)$$ (7)

$$H_{Y}=BERT\left(Y\right)$$ (8)

$$BERTScore\left(X,Y\right)=\frac{H_{X}^{T}H_{Y}}{|\left|H_{X}\right||\cdot |\left|H_{Y}\right||}$$ (9)

Fluency is evaluated using Perplexity (PPL), which measures the likelihood of a sentence based on word probabilities. Lower PPL values indicate better language model performance. In this study, the Chinese-based GPT-2 is used to calculate PPL, with higher predicted probabilities reflecting greater fluency.

$$\;PPL\left(X\right)={\left(P\left(w_{1}w_{2}\ldots w_{N}\right)\right)}^{-\frac{1}{N}}=\sqrt[{N}]{\prod_{i=1}^{N}\frac{1}{p(w_{i}|w_{1}w_{2}\ldots w_{i-1})}}$$ (10)

$$p\left(w_{i}|w_{1}w_{2}\ldots w_{i-1}\right)=M(w_{1}w_{2}\ldots w_{i-1};\theta )$$ (11)

Generative diversity, encompassing both divergence from the original utterance and variety among generated paraphrases, is evaluated using two complementary metrics:

- **iBLEU** [37]: quantifies divergence by rewarding overlap with the reference *Y* while penalizing overlap with the source *X*.
- **P-BLEU** [38]: measures richness as the average pairwise BLEU among the *k* generated paraphrases.

For an original utterance *X*, target reference *Y*, and *k* generated paraphrases $\left\{Y_{1},Y_{2},\ldots ,Y_{k}\right\}$, we define:

$$\text{iBLEU}(X,Y,\{Y_{i}{\}}_{i=1}^{k})\;=\;\frac{1}{k}\sum_{i=1}^{k}\left[\;\alpha \;\text{BLEU}(Y,Y_{i})\;-\;(1-\alpha )\;\text{BLEU}(X,Y_{i})\right]$$ (12)

P-BLEU({Yi}i=1k)=1k(k−1)∑i=1k∑j=1j≠ikBLEU(Yi,Yj) (13)

Here, α∈[0,1] balances semantic fidelity and novelty in iBLEU, and lower P-BLEU scores indicate greater internal diversity among the paraphrases. Together, these metrics offer a picture of generative diversity: iBLEU assesses each paraphrase’s trade-off between adequacy and novelty relative to the source and reference, while P-BLEU captures the overall spread of the model’s paraphrasing space by quantifying how similarly its outputs relate to each other.

### 5.2 Contrastive experiment

#### 5.2.1 ContraGAN contrastive experiment.

For supervised learning models, experiments utilized LCQMC (small-scale) and Phoenix (large-scale) datasets under low (*N* = 5) and high (*N* = 15) generation scenarios. Training employed a batch size of 64 with a maximum sequence length of 20 tokens. The Adam optimizer ($\beta_{1}=0.9,\beta_{2}=0.995$) was used, with ContraGAN’s initial learning rate set to $1\times 10^{-5}$, threshold *T* = 0.4, loss ratio 0.4, and smoothing parameter ε=0.1. LaserTagger was fine-tuned on RoBERTa-base with a lexicon size of 800, FELIX on GAU-alpha, and HRQ-VAE on Chinese BERT-base with a hidden variable dimension of 16. Default settings were used for other parameters.

Performance comparisons of ContraGAN and benchmark models under both scenarios are detailed in Table 5 and 6. Duplicate paraphrases were removed, and the best results are highlighted in bold, with metric directions indicated by arrows.

**Table 5:** Experiments on supervised text repetition generation in small generation number scenarios.

Dataset	Model	BERTScore↑	PPL↓	iBLEU↑	P-BLEU↓
LCQMC	T5	80.83	58.25	31.74	67.67
	LaserTagger	81.24	59.29	32.24	81.75
	FELIX	**82.15**	60.21	34.52	78.32
	BART	81.02	58.90	34.42	75.16
	HRQ-VAE	81.94	**57.62**	**35.53**	73.08
	**ContraGAN**	81.56	57.97	35.10	**64.23**
Phoenix	T5	77.54	59.12	33.21	63.23
	LaserTagger	80.31	59.73	34.85	78.57
	FELIX	**81.23**	60.48	35.74	75.33
	BART	80.11	59.62	35.90	71.56
	HRQ-VAE	79.90	59.05	**36.78**	70.89
	**ContraGAN**	80.38	**58.44**	36.11	**60.10**

**Table 6:** Experiments on supervised text repetition generation in big generation number scenarios.

Dataset	Model	BERTScore ↑	PPL ↓	iBLEU ↑	P-BLEU ↓
LCQMC	T5	79.76	60.31	31.68	66.91
	LaserTagger	80.20	60.73	32.28	79.75
	FELIX	80.95	61.44	34.60	77.53
	BART	81.12	59.45	35.10	74.68
	HRQ-VAE	80.68	59.69	35.55	72.38
	**ContraGAN**	**81.41**	**58.12**	**35.92**	**63.57**
Phoenix	T5	78.39	61.02	33.18	62.55
	LaserTagger	79.18	61.85	34.97	77.35
	FELIX	80.06	62.64	35.84	74.62
	BART	79.87	60.80	35.62	71.24
	HRQ-VAE	79.77	60.10	36.41	69.86
	**ContraGAN**	**80.23**	**58.63**	**36.49**	**60.07**

Table 5 and 6 demonstrate ContraGAN’s consistent superiority over baseline models across small and large generation scenarios. On LCQMC in small-sample scenarios (Table 5), ContraGAN surpasses T5 with improvements of 0.73 in BERTScore, 0.28 in PPL, 3.36 in iBLEU, and 3.44 in P-BLEU. It also achieves lower PPL and P-BLEU than LaserTagger and FELIX, while maintaining competitive iBLEU. Although HRQ-VAE shows higher iBLEU, ContraGAN delivers significantly lower P-BLEU (64.23), reflecting superior diversity.

In large-sample scenarios (Table 6), ContraGAN maintains top performance with the highest BERTScore (81.41) and lowest PPL (58.12) on LCQMC, alongside competitive iBLEU and the lowest P-BLEU (63.57). These results highlight ContraGAN’s balance between fidelity, fluency, and diversity, validating its effectiveness in text generation tasks.

#### 5.2.2 ContraMetrics contrastive experiment.

Experiments were conducted on BQ-Corpus and PAWS-X under unsupervised settings. Sequence lengths were set to 40 and 60, with a batch size of 64, and the Adam optimizer ($\beta_{1}=0.9$, $\beta_{2}=0.995$) was employed at a learning rate of $1\times 10^{-5}$. The ContraMetrics loss ratio *α* was 0.4, and SeqGAN utilized a pre-trained T5 generator. ContraMetrics applied a multi-indicator strategy based on BERTScore, PPL, and Self-BLEU [39] for positive and negative sample mining, with thresholds detailed in Table 7.

**Table 7:** Multi-indicator strategy threshold setting.

Indicator	*T*_*p*_	*T*_*N*_
BERTScore↑	75	60
PPL↓	60	75
Self-BLEU↑	50	35

Keyword prompt-based pre-training on LCQMC extracted up to three keywords per sample using LAC, formatted as "Text Repeat:". Table 8 and 9 compare ContraMetrics with baselines under small (*N* = 5) and large (*N* = 15) generation settings.

**Table 8:** Experiments on unsupervised text repetition generation in small generation number scenarios.

Dataset	Model	BERTScore ↑	PPL ↓	P-BLEU ↓
BQ-Corpus	DSS-VAE	**72.73**	64.28	77.86
	STVAE	71.64	64.84	66.15
	SeqGAN	72.56	65.49	63.13
	PEGASUS	71.88	64.59	65.23
	DPGAN	71.82	65.64	62.08
	**ContraMetrics**	72.26	**64.07**	**61.32**
Phoenix	DSS-VAE	**68.66**	71.12	73.31
	STVAE	66.59	72.47	59.98
	SeqGAN	68.33	72.56	58.69
	PEGASUS	67.98	71.08	61.62
	DPGAN	67.15	72.94	57.50
	**ContraMetrics**	68.05	**70.51**	**55.33**

**Table 9:** Experiments on unsupervised text repetition generation in big generation number scenarios.

Dataset	Model	BERTScore ↑	PPL ↓	P-BLEU ↓
BQ-Corpus	DSS-VAE	71.31	65.24	76.36
	STVAE	70.08	66.30	64.25
	SeqGAN	72.04	66.78	62.21
	PEGASUS	71.50	65.38	63.87
	DPGAN	70.45	65.93	62.58
	**ContraMetrics**	**72.26**	**64.22**	**60.60**
Phoenix	DSS-VAE	67.25	71.25	72.15
	STVAE	65.68	73.84	58.43
	SeqGAN	68.02	73.02	58.72
	PEGASUS	67.18	71.65	59.73
	DPGAN	66.81	73.27	57.96
	**ContraMetrics**	**68.03**	**70.82**	**55.05**

Table 8 shows ContraMetrics achieves superior PPL and P-BLEU on both datasets, with P-BLEU reductions of at least 0.76 and 2.17, respectively. DSS-VAE leads in BERTScore but sacrifices diversity. STVAE improves diversity yet lags in semantic fidelity and diversity compared to ContraMetrics. SeqGAN’s marginally higher BERTScore reflects its tradeoff for fluency and diversity, with ContraMetrics outperforming in PPL by 1.42 and 2.05.

Table 9 highlights ContraMetrics’ consistent dominance across metrics in large-generation settings. Stable BERTScore and PPL validate its ability to generate high-quality, diverse paraphrases at scale.

**Fig 8:**
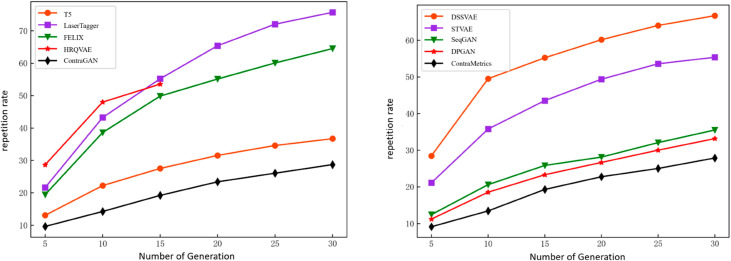
Comparison of repetition rates for text generation. (a) Under supervision, ContraGAN’s gradual repetition rate increase highlights its superior diversity. (b) In unsupervised settings, ContraMetrics maintains duplication rates below 20%, showing robust generative diversity, especially with more samples.

The comparison between two methods can be seen in Fig 8.

### 5.3 Ablation experiment and analysis

#### 5.3.1 Ablation experiment.

**Fig 9:**
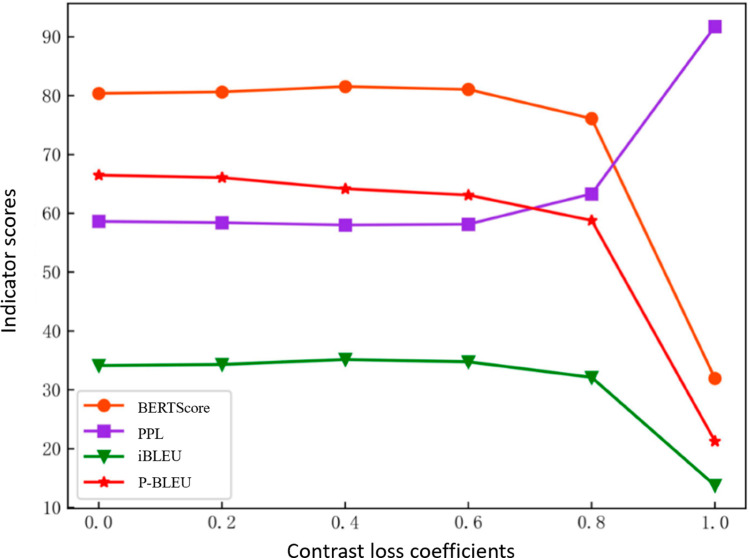
Illustration of model performance with different contrast loss coefficients.

1. **Contrastive Loss Ablation Experiment:** To evaluate the effectiveness of the encoder-decoder-based contrastive learning method proposed in this paper, an ablation experiment was designed focusing on contrastive loss. The models used include:
  - **Benchmark Model:** The pre-trained T5 model fine-tuned directly on LCQMC until convergence, serving as the baseline.
  - **ContraGAN-E:** This model applies encoder-based contrastive loss to the benchmark model, maintaining the structure of ContraGAN. The loss function used is $L\;=\;L_{\text{Encoder}}$.
  - **ContraGAN-D:** This model applies decoder-based contrastive loss to the benchmark model, also maintaining the structure of ContraGAN. The loss function used is $L\;=\;L_{\text{Decoder}}$.
  The results of the contrastive loss ablation experiments are summarized in Table 10.
  Observation of Table 10 shows that ContraGAN outperforms its sub-models across most metrics, except ContraGAN-E. The omission of decoder contrast loss in ContraGAN-E causes encoder-decoder mismatches, generating disordered outputs and reducing practical utility.
  ContraGAN achieves comprehensive improvements, notably a 0.92 increase in BERTScore and enhancements of 3.38 and 3.62 in iBLEU and P-BLEU over the baseline.
  ContraGAN-D shows slight declines in BERTScore and PPL but improves iBLEU and P-BLEU, indicating that its multi-positive-sample strategy enhances diversity and richness compared to T5’s single-objective training.
  Fig 9 illustrates the performance of the model at different values of coefficient *α*, which balances encoder and decoder losses. At α=0, the model becomes ContraGAN-D, while α=1 corresponds to ContraGAN-E. When α>0.8, performance drops sharply (except for P-BLEU) due to encoder loss dominance and a mismatched decoder. Conversely, at α=0.4, the model achieves optimal performance across all metrics by effectively balancing the two losses.
2. **Positive and Negative Sample Mining Strategy Ablation Experiment:** The baseline model is a pre-trained T5, using batch samples as negatives and target paraphrases as positives for self-contrastive learning. In ContraMetrics, target paraphrases are fixed positive samples for ablation. ContraGAN-N exclusively employs target paraphrases as positives. Results are shown in Table 11.
  From Table 11, the benchmark model, using batch samples as negatives, achieves the highest PPL but underperforms significantly on iBLEU and P-BLEU, indicating limited generative diversity. ContraMetrics improves by 0.23, 2.71, and 3.35 across metrics compared to the benchmark. ContraGAN further enhances performance with increases of 0.42, 3.49, and 5.10, excelling in diversity. ContraGAN-N, despite achieving the best BERTScore, lags in iBLEU and P-BLEU, reflecting its diversity limitations.
3. **Labeling Strategy Ablation Experiment:** The baseline model in these experiments is a simplified ContraGAN without additional strategies. It is compared with two variants: one using label smoothing and the other using pseudo-labeling. Results are shown in Table 12.
  The results show that the pseudo-labeling strategy substantially outperforms the baseline. Label smoothing further refines the discriminator’s probability distribution, reducing noise and enabling smoother differentiation between positive and negative samples.
4. **Prompt Strategy Ablation Experiment:** The baseline for this experiment is the ContraMetrics model without prompts, compared to versions with general prompts and keyword-based prompts on the BQ-Corpus dataset. The results are summarized in Table 13.
  The results show that incorporating prompts into the model significantly improves BERTScore and PPL compared to the baseline, though there is a slight decline in P-BLEU. The use of keyword-based prompts enhances semantic fidelity and fluency, with a notable increase of 2.66 in BERTScore and 3.67 in PPL, but a decrease of 2.74 in P-BLEU. This trade-off seems justified when evaluating the model’s overall performance.

**Table 10:** Contrast loss ablation experiment.

Model	BERTScore↑	PPL↓	iBLEU↑	P-BLEU↓
Benchmark Model	80.62	58.45	31.78	67.80
ContraGAN-E	31.97	91.75	13.76	**21.30**
ContraGAN-D	80.39	58.63	34.12	66.48
ContraGAN	**81.54**	**58.02**	**35.16**	64.18

**Table 11:** Positive and negative sample mining strategies for ablation experiment.

Model	BERTScore ↑	PPL ↓	iBLEU ↑	P-BLEU ↓
Benchmark Model	81.14	**57.15**	31.61	69.41
ContraMetrics	81.37	58.33	34.32	66.06
ContraGAN-N	**81.61**	57.64	33.13	66.75
ContraGAN	81.56	57.97	**35.10**	**64.31**

**Table 12:** Labeling strategies ablation experiment.

Model	BERTScore↑	PPL↓	iBLEU↑	P-BLEU↓
Baseline Model	81.38	58.15	33.92	67.41
+ Label Smoothing	81.42	57.53	**34.32**	66.96
+ Pseudo-Labeling	**81.54**	**57.27**	34.25	**65.67**

**Table 13:** Keyword-based prompts ablation experiment.

Model	BERTScore↑	PPL↓	P-BLEU↓
Baseline Model	69.46	67.92	**58.31**
+ Prompts	69.64	67.48	58.88
+ Keyword-based Prompts	**72.12**	**64.25**	61.05

### 5.4 Sample analysis

HRQ-VAE primarily focuses on grammatical alterations, sometimes leading to semantic contradictions, while ContraGAN performs more comprehensive transformations, including both grammatical restructuring and word substitution, while preserving semantics more effectively.

**Fig 10:**
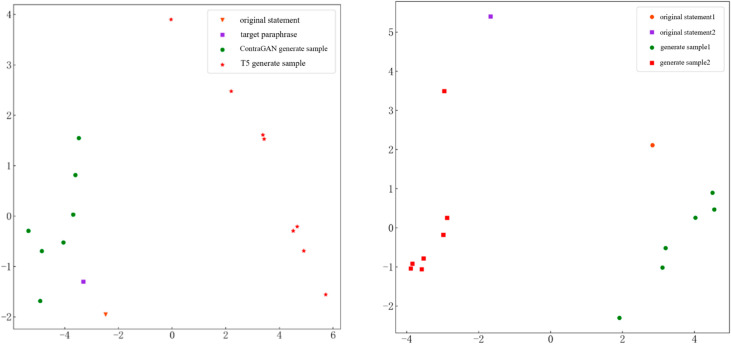
Scatterplot of feature vectors to generate recapitulation. (a) ContraGAN achieves a balanced distribution, enhancing paraphrase diversity while preserving semantic fidelity for different models. (b) Generated samples remain close to original utterances, minimizing inter-statement confusion for different statements.

The outputs of the models were sampled and visualized on a 2D plane to assess their practical effectiveness. For both T5 and ContraGAN, *N* = 10 outputs were generated to eliminate repetitions. The recapitulation pairs and generated samples were encoded into feature vectors, followed by dimensionality reduction using principal component analysis (PCA) for visualization. Results from varying repetition pairs were visualized using the same technique. The visualized outputs can be seen in Fig 10.

### 5.5 Error analysis

While the proposed ContraGAN and ContraMetrics models perform well overall, the following key issues need attention:

1. **Training Time:** The use of indefinite positive and negative samples in contrastive learning reduces parallelism, increasing training time.
2. **Performance in Small Generation Scenarios:** Models show limited advantages in small generation scenarios, particularly in semantic fidelity.
3. **Diversity of Generated Text:** Despite good overall diversity, some cases show insufficient variety, especially with smaller generation numbers. Improved generation strategies are needed to avoid repetition.

## 6 Conclusions

This paper presents a self-contrastive learning framework addressing core challenges in paraphrase generation, including exposure bias, model degradation, and insufficient diversity. The proposed ContraGAN and ContraMetrics models demonstrate significant advancements in both supervised and unsupervised scenarios by leveraging self-generated samples and keyword-guided prompts.Experimental results on benchmark datasets confirm substantial improvements, achieving notable gains of 0.46 in BERTScore, 1.57 in PPL, 0.37 in iBLEU, and 3.34 in P-BLEU over state-of-the-art methods. These findings validate the models’ capability to deliver fluent, diverse, and semantically faithful paraphrases, setting a new standard for text generation tasks.

Future work will prioritize enhancing efficiency and adaptability. Addressing the extended training time caused by unlimited sampling, we will explore fixed-sample strategies to streamline training. Furthermore, improving model robustness in low-generation scenarios through advanced language model-based discriminators will be a key focus. These efforts aim to further solidify the applicability and scalability of the proposed methods across diverse NLP applications.
